# Structures, Bonding and Sensor Properties of Some Alkaline *o*-Phthalatocuprates

**DOI:** 10.3390/ma14195548

**Published:** 2021-09-24

**Authors:** Sergey V. Gladnev, Mikhail V. Grigoryev, Mariya A. Kryukova, Evgenia M. Khairullina, Ilya I. Tumkin, Nikita A. Bogachev, Andrey S. Mereshchenko, Mikhail Y. Skripkin

**Affiliations:** Institute of Chemistry, Saint-Petersburg State University, 7/9 Universitetskaya Emb., 199034 St. Petersburg, Russia; st069020@student.spbu.ru (S.V.G.); mikegrig@inbox.ru (M.V.G.); m.a.kryukova@spbu.ru (M.A.K.); e.khairullina@spbu.ru (E.M.K.); i.i.tumkin@spbu.ru (I.I.T.); n.a.bogachev@spbu.ru (N.A.B.); a.mereshchenko@spbu.ru (A.S.M.)

**Keywords:** alkaline *o*-phthalatocuprates, crystal structure, vibrational spectra, force constants, electrochemical sensors, D-glucose, paracetamol, dopamine

## Abstract

Comprehensive study of the structure and bonding of disodium, dipotassium and diammonium di-*o*-phthalatocuprates(**II**) dihydrates has been undertaken. The crystal structure of ammonium *o*-phthalatocuprate has been determined. The identity of structures of phthalatocuprate chains in potassium and ammonium salts has been revealed. Vibrational spectra of all three compounds have been recorded, and the assignment of vibrational bands has been made. Force field calculations have shown a minor effect of outer-sphere cations (Na^+^, K^+^, NH_4_^+^) on both intraligand (C-O) and metal–ligand bond strengths. Synthesized compounds have been tested as electrochemical sensors on D-glucose, dopamine and paracetamol. Their sensitivity to analytes varied in the order of Na^+^ > K^+^ > NH_4_^+^. This effect has been explained by the more pronounced steric hindrance of copper ions in potassium and ammonium salts.

## 1. Introduction

Copper-based metal–organic frameworks (MOFs) with polycarboxylate linkers have recently attracted attention in the field of electrochemical sensors for the detection of different analytes [1,2,3,4] due to their potential for increasing electrode/electrolyte interfaces and reducing mass consumption by tuning single atomic metal centers. For example, Cu(BTC) MOF (BTC: trimesic acid) has shown peroxidase-like activity for electrochemical detection of H_2_O_2_ [5] and catechol [6], respectively. Cu-based MOFs, consisting of Cu^2+^ ions and ligands such as TPA (TPA: terephthaic acid) and BTC, have shown good electrochemical properties and excellent catalytic performance in electrochemical sensors for the detection of analytes such as glucose, H_2_O_2_, etc. [7,8]. Though numerous studies have been conducted in this field, there are mostly only empirical observations of electrocatalitical activity towards target analytes [9,10], and the factors affecting the sensor properties of the different types of materials with similar structures have not yet been fully identified. In this regard, in this work the effect of an outer-sphere cation (Na^+^, K^+^, NH_4_^+^) on structure and bonding in alkaline di-*o*-phthalatocuprate dihydrates was studied in detail to reveal the major factors that determine sensor activity of phthalatocuprates towards D-glucose.

## 2. Materials and Methods

### 2.1. Reagents

Copper(II) Carbonate Basic (>95%), Potassium Hydrogen Phthalate (>99.95%), Phthalic Acid (>98%), Sodium Hydroxide (>99.5%), D-Glucose, Paracetamol, Dopamine Hydrochloride (all—European Pharmacopoeia (EP) Reference Standard), PBS buffer, Nafion (5 wt% solution) and Ammonia (32% aqueous solution) were purchased from Sigma-Aldrich Ltd. (Darmstadt, Germany), Sodium Hydrogen Phthalate (> 99%) was supplied by Advanced Technology & Industrial Co., Ltd. (Hong Kong, China). All the reagents were used without further purification.

### 2.2. Synthesis of Alkaline di-o-Phthalatocuprates(**II**) Dihydrates

Disodium(**I**) and dipotassium(**II**) di*-o*-phthalatocuprates(**II**) dihydrates have been synthesized according to the method described in [11]. Copper(**II**) carbonate basic was dissolved in an aqueous solution containing a stoichiometric amount of alkaline hydrogen phthalate. As no ammonium hydrogen phthalate was available, this was prepared in situ simply by mixing equimolar amounts of ammonia and phthalic acid, and then stoichiometric amount of copper carbonate basic was added under stirring to obtain diammonium(**III**) di-*o*-phthalatocuprate(**II**) dihydrate. The solutions were allowed to stay at room temperature for slow evaporation; as a result, blue elongated prismatic crystals suitable for X-ray analysis were obtained for all three samples.

### 2.3. XRD Characterization

**Figure 1:**
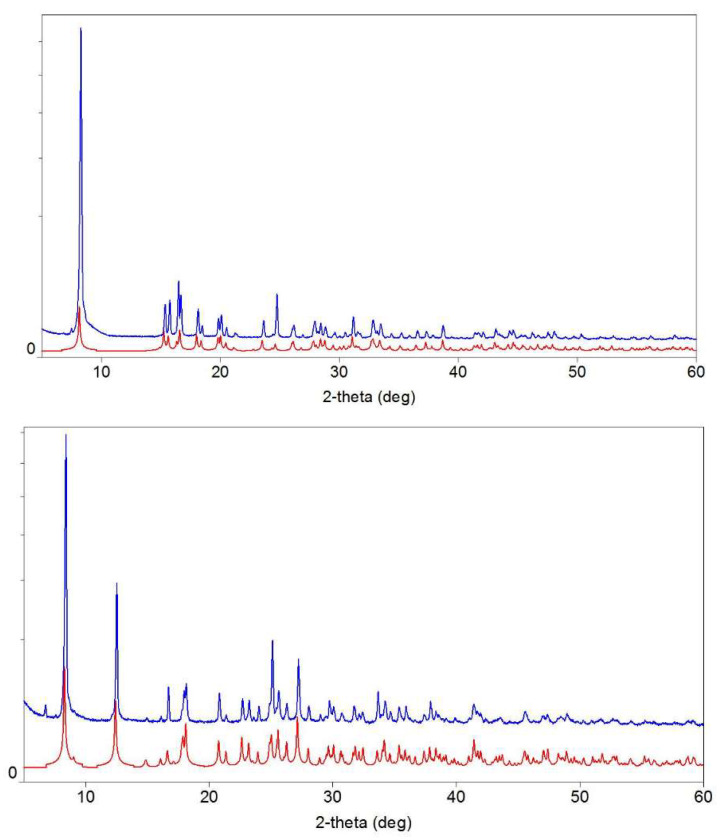
Comparison of powder diffraction pattern for synthesized samples (blue) with those calculated from single crystal X-ray data for disodium (CCDC number 1216797) and dipotassium (CCDC number 1200201) phthalatocuprates (red): **top**—compound **I**, **bottom**—compound **II**.

**Figure 2:**
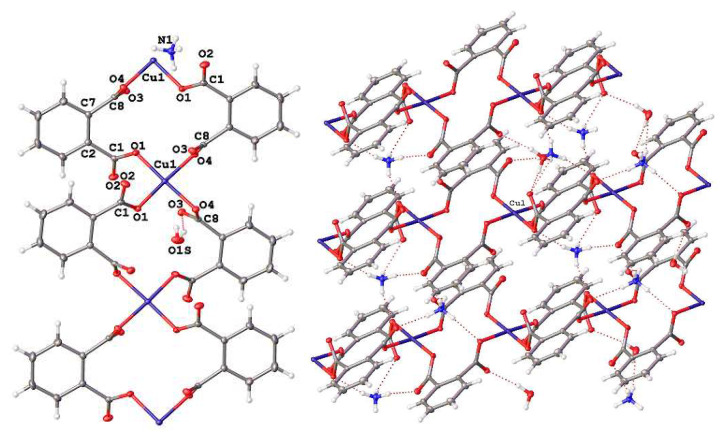
Coordination of *o*-phthalate ligands to copper ion (left) and crystal packing (right) in **III**. Hydrogen bonds are presented as dotted lines.

X-ray powder diffraction (XRD) measurements were performed on a D2 Phaser (Bruker, Billerica, MA, USA) X-ray diffractometer using Cu Kα radiation (λ = 1.54056 Å). Experimental powder fiffractograms are compared with calculated ones at Figure 1. As no crystallographic data for III are available so far, single-crystal X-ray diffraction analysis was undertaken. Data were collected using Agilent Technologies «Xcalibur» diffractometer K using a monochromatic radiation source (Mo*Kα* radiation, λ = 0.71073). The structure was solved using the ShelXT [12] structure solution program by Intrinsic Phasing and refined with the ShelXL [12] incorporated in the Olex2 refinement package [13]. Empirical absorption correction was applied in the CrysAlisPro (Agilent Technologies, Santa Clara, CA, USA, 2014) program complex using spherical harmonics, implemented in SCALE3 ABSPACK scaling algorithm. The carbon-bound H atoms were placed in calculated positions and were included in the refinement in the riding model approximation, with U*_iso_*(H) set to 1.2U*_eq_*(C) and C–H 0.93 Å for the CH groups, U*_iso_*(H) set to 1.6U*_eq_*(N) and N–H in the corresponding bond lengths for the NH groups, and U*_iso_*(H) set to 1.5U*_eq_*(O) and O–H 0.85 Å for the OH groups. The crystallographic parameters and the structure refinement statistics for III at T = 293(2) K are as follows: C_16_H_20_CuN_2_O_10_, M_w_ = 463.88 g/mol, space grou I2/a, a = 7.9889(3) Å, b = 21.2012(8) Å, c = 11.5131(4) Å, ɑ = 90, β = 107.398(4), γ = 90, V = 1860.81(12) Å3, Z = 4 (Z’= 1), T = 293(2) K, μ (MoKα) = 1.234 mm^−1^, Dcalc = 1.656 g/cm^3^, 20,109 reflections measured (5.678° ≤ 2Θ ≤ 64.758°), 3093 unique (R_int_ = 0.0476, R_sigma_ = 0.0287) which were used in all calculations, ρ_calc_ = 1.656 g/cm^3^, F (000) = 956.0. The final R1 was 0.0280 (I > 2σ(I)) and wR2 was 0.0917 [14]. Fractional atomic coordinates, equivalent isotropic displacement parameters, anisotropic displacement parameters, complete lists of bond lengths and bond angles are summarized in Tables S1–S6 in Supporting Information. The structure is presented at Figure 2 and the selected distances are reported in Table 1 and Table 2.

### 2.4. Vibrational Spectroscopy

**Figure 3:**
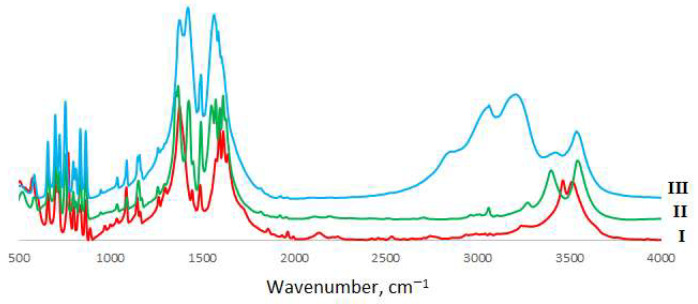
Mid-IR spectra of *o*-phthalatocuprates.

**Figure 4:**
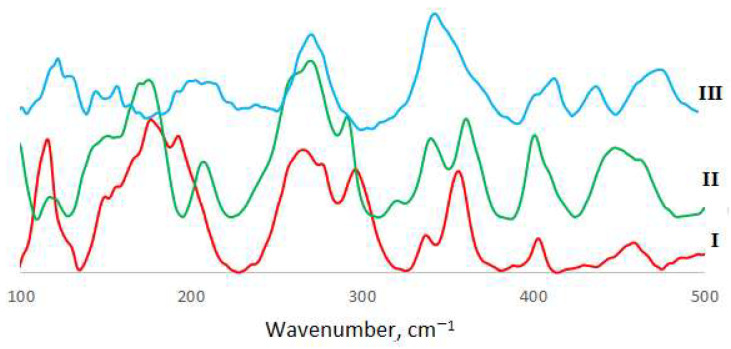
Far-IR spectra of *o*-phthalatocuprates.

**Figure 5:**
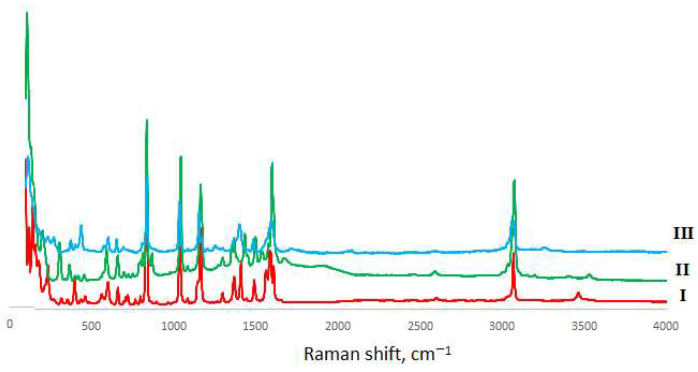
Raman spectra of *o*-phthalatocuprates.

Mid-IR spectra have been recorded in the 400–4000 cm^−1^ spectral region by a Nicolet 8700 (Thermo Scientific, Waltham, MA, USA) spectrometer by means of the ATR technique. Kramers–Kronig correction was applied to eliminate ATR distortions. Far-IR measurements have been performed in the spectral range 65–700 cm^−1^ at the same equipment. The diffuse reflectance (DRFTS) technique was applied. Raman spectra were recorded by means of SENTERRA (Bruker, Billerica, MA, USA) express Raman spectrometer equipped with Peltier cooled CCD detector (Bruker, Billerica, MA, USA) at 488 nm excitation. For all the spectral measurements 128 scans were performed and averaged, 4 cm^−1^ spectral resolution was applied. All spectra were measured at an ambient temperature. Baseline correction and deconvolution of spectral contours were made using the GRAMS32 package (Galactic Industries, Salem, NH, USA). The experimental spectra are shown in Figure 3, Figure 4 and Figure 5, with experimental frequencies summarized in Table S7.

Vibrational bands that appear only in the spectra of compounds **I**–**III** and are absent in spectra of neat acid were assigned to copper–oxygen stretchings. They were observed as weak to medium intensity bands at 306–307 cm^−1^ (Raman) and 317–319 cm^−1^ (IR). As the geometry of the copper ion coordination sphere is very close to the square planar, we assigned the Raman active frequency to symmetric and another to asymmetric stretching.

To understand in more detail the bonding in alkaline phthalatocuprates, we have undertaken force-field calculations. Though the force constant reflects the slope of the potential energy surface near equilibrium points, for similar molecules, the close relationship in trends for stretching force constants and bond energies is well known. C_6_H_4_(COOCu)_2_ and Cu(OC(O)C)_4_ moieties were considered to analyze both intraligand bonding and the coordination effect. Wilson’s GF matrix method was used for the calculation of vibrational frequencies using a symmetrized valence force field. The PC-based program package, written in FORTRAN, is developed by J. Mink and L. Mink [15]. Initial force constants were adopted from Colombo et al. [16] and refined to obtain a good coincidence of calculated and experimental data (within 1–2 cm^−1^). The average experimental frequencies were taken because of the interligand interactions. The experimental and calculated vibrational frequencies of the carboxylate group together with potential energy distribution and refined force constants for this moiety are summarized in Table 3 and Table 4 below.

### 2.5. Electrochemical Measurements

The electrochemical studies (CorrTest CS300, OhmLiberScience, Saint-Petersburg, Russia) were conducted in a standard three-electrode cell: modified GCE (d = 3 mm) was used as the working electrode, a platinum mesh as a counter electrode and an Ag/AgCl as reference electrode. Modified GCEs were prepared by sequential drop-casting of 10 µL water-based suspension of alkaline di-*o*-phthalatocuprates (1 mg/mL) and 5 µL of Nafion solution (0.02 wt%); after that, modified electrodes were dried under ambient conditions for further electrochemical experiments. The cyclic voltammetry measurements (CV) were performed within the potential range −0.2–0.8 V vs. Ag/AgCl with scan rate of 50 mV/s. The differential pulse voltammograms (DPV) were recorded within the potential range 0–0.6 V, with an amplitude of 0.05 V and step potential of 0.004 V. The solutions of analytes (Gl—glucose, DA—dopamine, AP—paracetamol) of different concentrations were added to the corresponding background solutions (0.1 M sodium hydroxide for Gl and 0.1 M PBS for DA and PA).

## 3. Results and Discussion

### 3.1. Crystal Structure

The powder diffraction patterns of synthesized **I** and **II** compounds (Figure 1) confirmed their phase purity [11]. As ammonium salt (**III**) was obtained for the first time, single-crystal diffraction study was undertaken. The results have shown that the copper ion in this compound is surrounded by four *o*-phthalate ligands in a nearly square-planar coordination (Figure 2), similar to that in compounds **I** and **II**. Copper–oxygen bond lengths in **III** are equal to 1.9474(8) Å and 2.0060(8) Å that is rather similar to those in potassium compound **II** (1.930(5) Å and 1.999(5) Å [11]) and a little higher than in sodium salt **I** (1.936(3) Å and 1.969(4) Å [11]). These bond distances are close to those found in other square-planar copper complexes with o-phthalate ligands (1.9336–1.9935 [17,18]). Bond distances in the benzene ring remain the same in all three compounds. As for carboxylate groups, some shortening of C-O bonds is observed in the order of K-NH_4_-Na.

The crystal packing in the compounds under study is somehow different. In all three substances, copper ions together with *o*-phthalatoligands form 1D polymeric chains. Water molecules and alkaline cations (or NH_4_^+^) are located between adjacent chains and associated 1D chains into 2D layers via hydrogen bonds or electrostatic interactions with phthalate-ions (Figure 2). In (**I**), the outer-sphere cation (Na^+^) is small enough to be able to be inserted into the hollows of the crossed linear polymeric chains, whereas the increase in ion size prevents this insertion and, as a result, potassium and ammonium ions interpose between adjacent zigzag chains. That results in less distance between the copper ion and non-coordinated oxygen ions from carboxylate groups (2.533 (5) Å and 2.5474 (5) Å in **II** and **III** vs. 2.755 (5) Å in **I**). One can mention that despite the ammonium ion being capable of forming hydrogen bonds with oxygen atoms of carboxylic groups (see Figure S1), which does not effect on the crystal packing in compound **III**—the effect of ionic size dominates.

### 3.2. Vibrational Spectroscopy

Vibrational spectroscopy is a powerful tool that enables us to compare bond strengths in chemical compounds without destroying the sample. The assignments of vibrational bands for phthalic acid, the *o*-phthalate ion and the hydrogen *o*-phthalate ion have been undertaken by several researchers: Colombo et al. [16], Arenas et al. [19,20], Martinez et al. [21] and Loring et al. [22]. However, Arenas and Lorig only took into account bands above 1000 cm^−1^ and Martinez has not analyzed C-H stretchings and carboxylic group vibrations. Therefore, our analysis was based mostly on that performed by Colombo as all vibrational bands of phthalic acid have been taken into account, and the assignment was also supported by quantum chemical calculations and by force field calculations.

That results in values rather similar to those obtained for other benzenedicarboxylic acids (e.g., terephthalic acid and its derivatives [19,20,23,24,25]). We have only re-assigned an intense band at 832 cm^−1^ to totally symmetric ring-breathing vibrations instead of OH out-of-plane mode—breathing mode should be the strongest one in Raman spectra. The problem with intensity in the measurements of Colombo et al. [16] can originate from single-crystal (not powdered) Raman measurements. An increase in the number of vibrational modes for alkaline di-*o*-phthalatocuprates(II) is due to the increase in number of o-phthalate moieties in the unit cell and interaction between ligands. The complete list of vibrational frequencies and their assignment is given in Table S5.

The comparison of the spectra of *o*-phthalic acid and *o*-phthalatocuprates under study allowed us to assign the modes originating from the carboxylate group (as they are the most affected by coordination to metal ions instead of hydrogen) and metal–ligand modes. Strong to medium intensity bands in the region of 1600–1640 cm^−1^ were assigned to the C=O stretching mode; lower-frequency modes are more intense in Raman and correspond to in-phase, whereas higher-frequency bonds are more intense in IR and corresponds to out-of-phase stretching. The positions of these bands coincide well with those in neat o-phthalic acid (1637 and 1640 cm^−1^). Weak bands at 1250–1270 cm^−1^ were assigned to C-O stretchings (1263 and 1280 cm^−1^ in neat acid); a lower frequency compared to acid can result both from the decrease in bond strength and also from the mass difference between hydrogen and copper atoms bonded with the C-O group. The comparison of these data with those for metal ion complexes with terephthalate ligands studied earlier [24,25] shows far less splitting of carboxylate group vibrational frequencies in o-phthalate complexes because of non-centrosymmetric position of CO_2_ groups.

CO_2_ deformations in complexes were found as medium intensity bands at 650–660 cm^−1^, and C-O twist as a weak to medium intensity band at 265–277 cm^−1^. As to the bands around 3500 cm^−1^, they can be attributed to OH stretching modes; the difference in H-bonding in **I** and **II**-**III** can be clearly observed from IR spectra.

As one can see from the obtained results, the force constants obtained are similar to those for phthalic acid. Some increase in C-O stretching force constants reflects a lower polarization effect of copper ions compared to hydrogen. As for copper–oxygen bond strengths, the results are rather similar to those for another complex with oxygen-donor ligands [26]. It should be mentioned that no obvious effect of outer-sphere cations on metal–ligand bond strengths is observed in *o*-phthalatocuprates, in contrast to that found in halide complexes [26,27]. The polymeric structure of complexes and large size of ligands leads to the smoothing of the effect of polarization ability of outer-sphere ions. Intraligand force constants vary to a greater extent, which is probably due to the difference in interaction of outer-sphere cations with the oxygen atoms of carboxylate groups [11].

### 3.3. Sensor Properties

The monitoring of various analytes in body fluids plays a crucial role in the diagnosis and treatment of many disorders. The changes in glucose level in human blood is closely related to diabetes; in turn, variations in neurotransmitter concentrations (such as DA) are associated with Parkinson disease, Huntington’s chorea, addiction, etc. [28,29]. In this regard, the selective determination of bioanalytes in the presence of the most commonly used drugs worldwide (for example AP) is of great importance not only for clinical practice, but also for biomedical and biochemical research. Electrochemical methods offer a unique combination of expensiveness, flexibility and high performance, and have a great potential in non-enzymatic detection of aforementioned analytes.

**Figure 6:**
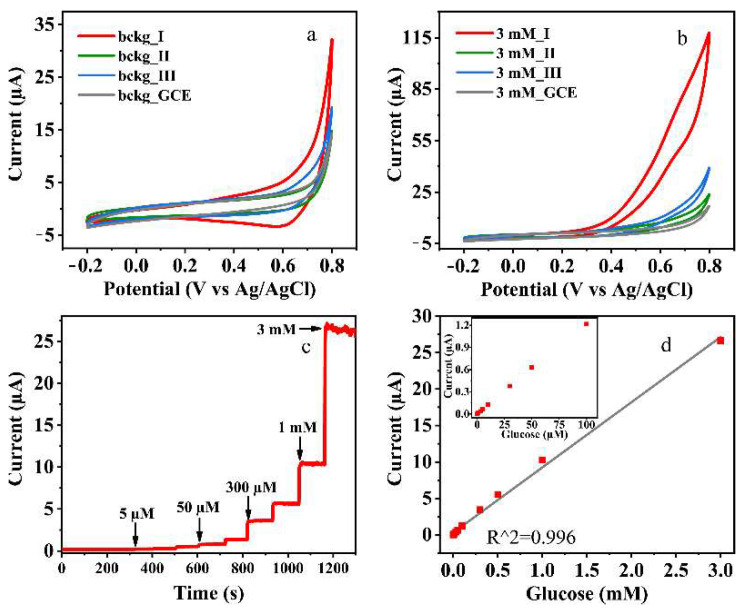
CV of alkaline *o*-phthalatocuprates in (**a**) background electrolytes and (**b**) in the presence of 3 mM of D-glucose. (**c**) Chronoamperogram of **I**/GCE recorded under potentiostatic conditions 0.55 V and (**d**) calibration curve for I obtained from CA data (Faraday current vs. [D-glucose]).

The electrocatalytic activities of the synthesized *o*-phthalatodicuprates towards D-glucose oxidation were investigated using CV and CA techniques (Figure 6). Figure 6a,b demonstrate that sensor response toward addition of 3 mM D-glucose is decreased in the order of **I** > **II** > **III**. Therefore, the sample **I** was chosen for further studies by chronoamperometry, and the typical CA curve recorded during consecutive additions of D-glucose aliquots to 0.1 M NaOH at potentials of 0.55 V presented on Figure 6c. The linear range of dependence of the Faraday current on the D-glucose concentration lies between 1 µM and 3 mM, wherein sensitivity and the limit of detection (LOD) were equal to 8.95 µA/mM and 0.26 µM, respectively. Thereby, sodium salt **I** exhibits sensor properties comparable with those of another copper-based MOFs known from the literature [30,31,32,33]. Table 5 presents the comparison between fabricated glucose sensor and other recently reported ones. Notably, the proposed electrode exibits comparable characteristics with a wide range of MOF-based sensors including complex composite materials.

This difference in sensor properties of the compounds under study cannot be explained by the difference in copper–ligand bonding: both metal–oxygen distances and stretching force constants do not differ so much. According to [9,30], the reaction mechanism involves two steps:

Cu(II) − MOF + OH^−^ = Cu(III) − MOF + e^−^ + H_2_O (1)

Cu(III) − MOF + Glucose = Cu(II) − MOF + Glucolactone,(2)

so, the availability of copper ion for nucleophilic attack can play a crucial role. Probably, the major factor affecting the sensor properties of these compounds is more steric hindrance of copper ion in compounds **II** and **III**, where the distance between the copper ion and non-coordinated oxygen ion of phthalate ligands is approximately 10% less compared to that in **I**. As for the difference in properties of potassium and ammonium salts that can result from a different kind of bonding between outer-sphere cations and the ligand—an electrostatic one in **II** and hydrogen bonding in **III**. The formation of hydrogen bond networks should prevent the penetration of the analyte into a complex and its interaction with the metal center that determines the activity according to the established mechanism of glucose oxidation on copper-based electrode materials [34,35].

**Figure 7:**
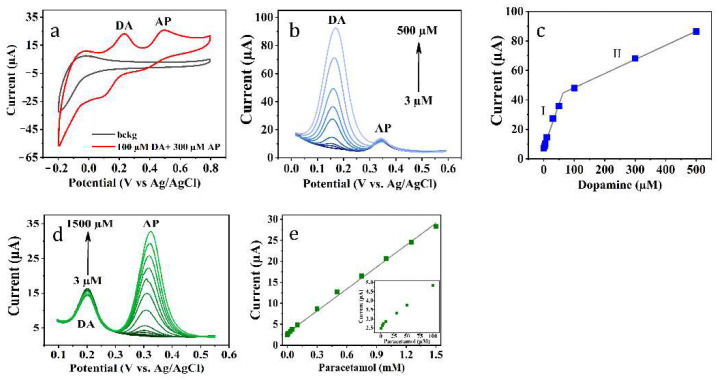
(**a**) CV of disodium di-*o*-phthalatocuprate–based electrode in background electrolyte and in presence of DA and AP; (**b**) DPVs of I/GCE recorded in mixture containing 50 µM AP with different concentrations of DA; (**d**) 20 µM DA with different concentrations of AP; the linear relationship between peak currents and the concentrations of (**c**) DA and (**e**)AP.

Furthermore, the determination of AP and neurotransmitter DA in binary mixtures was studied using CV and differential pulse voltammetry techniques (DPV). Figure 7a shows cyclic voltammograms of the **I**/GCE electrode in 0.1 M PBS (pH 7.4); as one can notice, there are two distinct peaks around 0.23 V and 0.49 V corresponding to the oxidation of DA and AP, respectively (Figure S2). DPVs for AP and DA at the sodium di-*o*-phthalatodicuprate–based electrode was obtained by changing the concentration of DA with a fixed concentration of AP (Figure 7b), and vice versa (Figure 7d). The current responses of both substrates increase linearly with their concentrations (Figure 7c,e). The linear regime of AP detection is provided within the range of 3–1500 µM, while there are two linear regions of DA concentration (3–60 µM and 60–500 µM). The calculated limits of detection were equal to 0.95 and 1.17 μM for DA and AP, respectively (LOD = 3S/b. Here, S is the standard deviation of ten blank measurements and b is the calibration curve slope). The analytical curve equations were found to be:

DA:

(I) I (µA) = 0.589 [DA] (µM) + 7.669

(II) I (µA) = 0.096 [DA] (µM) + 38.700

AP:

I (µA) = 0.017 [AP] (µM) + 2.951

## 4. Conclusions

In summary, the crystal structure of diammonium di-*o*-phthalatocuprate(II) dihydrate was determined for the first time. This structure is nearly identical to that of dipotassoum di-*o*-phthalatocuprate(II) dihydrate, which points to the domination of ionic size effect onto possible hydrogen bond formation in alkaline *o*-phthalatocuprates. The vibrational spectroscopic and force field study of disodium, dipotassium and diammonium di-*o*-phthalatocuprates has shown the minor dependence of copper–ligands in intraligand bond strengths on outer-sphere cations in contrast to the complexes with monoatomic ligands that can be explained by rigid polymeric structure of di-*o*-phthalatocuprates under study. Synthesized alkaline di-*o*-phthalatocuprates, especially disodium di-*o*-phthalatodicuprate, exhibit electrocatalytic activity towards a range of substrates, among them Gl, DA and AP. Better sensor properties of disodium di-*o*-phthalatocuprate are determined by less steric hindrance of copper ions in this compound compared to potassium and ammonium salts that enable the attack of the metal center by incoming ligands. The choosing of proper analytical methods and the optimisation of techniques allow one to fabricate universal electrode material for the selective detection of biosubstances of great importance with decent sensitivity.

## Figures and Tables

**Table 1:** Bond Lengths for **III**.

Atom	Atom	Length/Å	Atom	Atom	Length/Å
Cu1	O4 ^1^	2.0060(8)	O1	C1	1.2776(14)
Cu1	O4 ^2^	2.0060(8)	O3	C8	1.2523(13)
Cu1	O1 ^3^	1.9474(8)	O2	C1	1.2473(14)
Cu1	O1	1.9474(8)	C1	C2	1.5073(15)
O4	Cu1 ^1^	2.0060(8)	C8	C7	1.4989(15)
O4	C8	1.2758(13)			

Asymmetric units: ^1^ 3/2 − X, 3/2 − Y, 1/2 − Z; ^2^ X, 3/2 − Y, ½ + Z; ^3^ 3/2 − X, Y, 1 − Z.

**Table 2:** Comparison of selected bonds distances in compounds **I**–**III**.

Contact	Distance, Å		
	I	II	III
Cu-O1	1.969(4)	1.999(5)	2.0060(8)
Cu-O4	1.936(3)	1.930(5)	1.9474(8)
C1-O1	1.273(6)	1.263(6)	1.2776(14)
C1-O2	1.237(6)	1.253(6)	1.2473(14)
C8-O3	1.237(6)	1.221(6)	1.2523(13)
C8-O4	1.288(6)	1.296(6)	1.2758(13)

**Table 3:** Experimental and calculated vibrational frequencies (cm^−1^) of carboxylate moieties together with potential energy distribution (%) in **I**–**III**.

I		II		III		PED	Ass.
Exp.	Calc.	Exp.	Calc.	Exp.	Calc.		
1640	1639	1629	1629	1630	1630	75 ν(C=O), 24 ν(C-O)	ν_oop_(C=O)
1612	1613	1611	1611	1610	1611		ν_ip_(C=O)
1263	1263	1270	1268	1259	1269	63 ν(C-O), 21 δ(CO_2_)	ν_oop_(C-O)
1265	1263	1258	1260	1252	1260		ν_ip_(C-O)
660	660	661	660	658	657	68 δ(CO_2_), 13 ν(C-O)	δ_oop_(CO_2_)
653	654	651	652	653	653		δ_ip_(CO_2_)
317	317	319	319	318	317	70 ν(CuO), 30 δ(CuOC)	ν_a_(CuO)
305	305	307	307	306	306		ν_s_(CuO)
277	275	272	273	271	269	90 τ(CO)	τ_oop_(CO)
267	268	260	261	260	260		τ_ip_(CO)

**Table 4:** Refined force constants for **I**–**III**. Stretching force constants are given. 10^2^ N m^−1^, bending—10^−18^ N m.

Force Constant	I	II	III	Acid [16]
Stretch				
Cu-O	1.465	1.460	1.462	
C-O	6.022	6.071	6.057	5.93
C=O	8.409	8.621	8.616	8.5
C-C	2.503	2.541	2.539	2.6
Stretch-stretch				
Cu-O, Cu-O (trans)	−0.132	−0.167	−0.175	
C-O, Cu-O	0.116	0.125	0.112	
Bending				
CO_2_	1.654	1.578	1.589	1.65
CO—torsion	0.266	0.277	0.263	0.21

**Table 5:** Electrochemical performance of the sodium o-phthalatocuprate/GC electrode and similar materials for enzyme-free glucose sensing.

Electrode Material	Linear Range (µM)	LOD (µM)	Refs.
Cu-MOF/CNHs/GCE	0.25–1200	0.078	30
Multiplayer films of Cu-MOF/MWNTs	0.5–2340	0.4	9
Cu-in-ZIF-8	Up to 700	2.76	31
NiCu-MOF-6	20–4930	15	32
Ni@Cu-MOF	5–2500	1.67	33
Cu-MOF	10–3500	2.4	10
Sodium o-phthalatocuprate/GCE	1–3000	0.26	This work

## Data Availability

Data is contained within the article or supplementary material.

## Supplementary Materials

The following are available online at https://www.mdpi.com/article/10.3390/ma14195548/s1, Table S1: Crystal data and structure refinement for III, Table S2: Fractional Atomic Coordinates (×104) and Equivalent Isotropic Displacement Parameters (Å2 × 103) for III, Table S3: Anisotropic Displacement Parameters (Å2 × 103) for III, Table S4: Bond Lengths for III, Table S5: Bond Angles for III, Table S6: Hydrogen Atom Coordinates (Å × 104) and Isotropic Displacement Parameters (Å2 × 103) for III, Table S7: Vibrational Spectra of Alkaline o-Phthalatodicuprates and Their Assignment, Figure S1: Ammonium ion interaction with phthalate ions in III, Figure S2: Chemical transformations of (1) Gl, (2) DA, and (3) AP during the electrooxidation.
