# Supplementary material for: Structures, Bonding and Sensor Properties of Some Alkaline o-Phthalatocuprates
Source: Materials (Basel). 2021 Sep 24;14(19):5548. doi: 10.3390/ma14195548 (PMC8509265; doi:10.3390/ma14195548)
Supplement: Supplementary file 1 [file materials-14-05548-s001.zip › materials-1338874-supplementary.pdf]

Supplementary Materials

Structures, Bonding and Sensor Properties of Some Alkaline *o*-Phthalatocuprates

Sergey V. Gladnev, Mikhail V. Grigor’ev, Maria A. Kryukova, Evgenia M. Khairullina, Ilya I. Tumkin, Nikita A. Bogachev, Andrey S. Mereshchenko, Mikhail Y. Skripkin *

Contents

Table S1. Crystal data and structure refinement for **III** 3

Table S2. Fractional Atomic Coordinates (×10^4) and Equivalent Isotropic Displacement Parameters (Å^2×10^3) for **III** 4

Table S3. Anisotropic Displacement Parameters (Å^2×10^3) for **III** 4

Table S4. Bond Lengths for **III** 5

Table S5. Bond Angles for **III** 5

Table S6. Hydrogen Atom Coordinates (Å×10^4) and Isotropic Displacement Parameters (Å^2×10^3) for **III** 6

Table S7. Vibrational Spectra of Alkaline *o*-Phthalatodicuprates and Their Assignment 6

Figure S1. Ammonium ion interaction with phthalate ions in **III** 11

Figure S2. Chemical transformations of (1) Gl, (2) DA, and (3) AP during the electrooxidation 12

Table S1. Crystal data and structure refinement for **III**

Identification code in CCDC	2101455
Empirical formula	$C_{16}H_{20}CuN_2O_{10}$
Formula weight	463.88
Temperature/K	293(2)
Crystal system	monoclinic
Space group	I2/a
a/Å	7.9889(3)
b/Å	21.2012(8)
c/Å	11.5131(4)
α/°	90
β/°	107.398(4)
γ/°	90
Volume/Å^3	1860.81(12)
Z	4
ρ_{calc}g/cm^3	1.656
μ/mm^{-1}	1.234
F(000)	956.0
Crystal size/mm^3	0.5 × 0.3 × 0.2
Radiation	MoKα (λ = 0.71073)
2Θ range for data collection/°	5.678 to 64.758
Index ranges	$-11 \leq h \leq 12$, $-31 \leq k \leq 29$, $-17 \leq l \leq 16$
Reflections collected	20109
Independent reflections	3093 [R_{int} = 0.0476, R_{sigma} = 0.0287]
Data/restraints/parameters	3093/0/147
Goodness-of-fit on F^2	0.784
Final R indexes [I>=2σ (I)]	R_1 = 0.0280, wR_2 = 0.0917
Final R indexes [all data]	R_1 = 0.0311, wR_2 = 0.0966
Largest diff. peak/hole / e Å^{-3}	0.57/-0.46

Table S2. Fractional Atomic Coordinates (×10^4) and Equivalent Isotropic Displacement Parameters (Å^2×10^3) for **III**. U_{eq} is defined as 1/3 of of the trace of the orthogonalised U_{IJ} tensor.

**Atom**	***x***	***y***	***z***	**U(eq)**
Cu1	7500	7660.4(2)	5000	8.88(8)
O4	8083.2(10)	6649.3(4)	1248.3(7)	11.86(16)
O1	6849.5(11)	7021.1(4)	3729.4(7)	13.53(17)
O3	5243.6(10)	6832.7(4)	870.5(7)	13.80(16)
O2	4650.8(11)	6629.1(4)	4350.1(7)	17.28(18)
O1S	2208.5(12)	6762.4(5)	1484.7(8)	24.3(2)
N1	865.7(14)	6945.6(5)	3427.3(10)	15.56(19)
C1	5764.7(14)	6589.0(5)	3791.1(9)	11.7(2)
C8	6592.4(14)	6520.0(5)	1382.7(9)	10.67(19)
C2	5924.1(13)	5976.9(5)	3166.6(9)	11.9(2)
C7	6422.0(13)	5946.3(5)	2099.7(9)	11.7(2)
C6	6603.0(16)	5358.1(6)	1601.4(11)	17.3(2)
C3	5661.6(16)	5418.2(6)	3732.3(10)	16.7(2)
C4	5876.5(17)	4835.7(6)	3241.0(12)	20.8(2)
C5	6337.3(17)	4803.3(6)	2174.8(12)	21.0(2)

Table S3. Anisotropic Displacement Parameters (Å^2×10^3) for **III**. The Anisotropic displacement factor exponent takes the form: $-2\pi^2[h^2a^{*2}U_{11}+2hka^*b^*U_{12}+\ldots]$.

**Atom**	**U_{11}**	**U_{22}**	**U_{33}**	**U_{23}**	**U_{13}**	**U_{12}**
Cu1	7.14(11)	11.36(12)	8.59(12)	0	3.03(8)	0
O4	9.4(3)	15.3(4)	11.8(3)	1.6(3)	4.5(3)	-0.2(3)
O1	16.0(4)	15.0(4)	10.9(3)	-2.1(3)	6.1(3)	-4.3(3)
O3	10.1(3)	17.7(4)	13.9(4)	3.2(3)	4.0(3)	2.2(3)
O2	13.4(4)	25.6(4)	14.8(4)	-4.3(3)	7.3(3)	-2.1(3)
O1S	13.3(4)	43.3(6)	16.1(4)	-0.1(4)	4.0(3)	4.8(4)
N1	15.8(5)	17.9(5)	13.1(4)	-0.2(3)	4.5(4)	1.6(4)
C1	10.8(4)	14.4(5)	9.4(4)	0.7(3)	2.3(3)	-0.8(3)

C8	10.6(4)	13.1(5)	8.5(4)	-0.7(3)	3.2(3)	-0.8(3)
C2	10.0(4)	13.2(5)	12.4(4)	-0.2(3)	3.5(4)	-1.7(3)
C7	9.4(4)	12.8(5)	12.8(4)	0.7(3)	3.3(4)	-0.5(3)
C6	19.4(5)	16.3(5)	18.8(5)	-1.5(4)	9.8(4)	1.2(4)
C3	17.7(5)	17.8(5)	15.4(5)	2.7(4)	6.3(4)	-3.1(4)
C4	23.9(6)	13.6(5)	26.1(6)	4.6(4)	9.2(5)	-3.1(4)
C5	24.0(6)	12.7(5)	28.4(6)	-1.4(4)	11.0(5)	0.8(4)

Table S4. Bond Lengths for **III**.

**Atom**	**Atom**	**Length/Å**	**Atom**	**Atom**	**Length/Å**
Cu1	O4^{1}	2.0060(8)	C1	C2	1.5073(15)
Cu1	O4^{2}	2.0060(8)	C8	C7	1.4989(15)
Cu1	O1^{3}	1.9474(8)	C2	C7	1.4012(15)
Cu1	O1	1.9474(8)	C2	C3	1.3976(16)
O4	Cu1^{1}	2.0060(8)	C7	C6	1.3982(15)
O4	C8	1.2758(13)	C6	C5	1.3957(17)
O1	C1	1.2776(14)	C3	C4	1.3901(17)
O3	C8	1.2523(13)	C4	C5	1.3847(19)
O2	C1	1.2473(14)			

^{1}3/2-X,3/2-Y,1/2-Z; ^{2}+X,3/2-Y,1/2+Z; ^{3}3/2-X,+Y,1-Z

Table S5. Bond Angles for **III**.

**Atom**	**Atom**	**Atom**	**Angle/°**	**Atom**	**Atom**	**Atom**	**Angle/°**
O4^{1}	Cu1	O4^{2}	86.30(5)	O3	C8	O4	122.15(10)
O1^{3}	Cu1	O4^{1}	90.97(4)	O3	C8	C7	118.80(9)
O1	Cu1	O4^{2}	90.97(4)	C7	C2	C1	123.01(9)
O1	Cu1	O4^{1}	176.96(3)	C3	C2	C1	117.49(10)
O1^{3}	Cu1	O4^{2}	176.96(3)	C3	C2	C7	119.39(10)
O1	Cu1	O1^{3}	91.79(5)	C2	C7	C8	122.78(9)
C8	O4	Cu1^{2}	102.62(6)	C6	C7	C8	117.41(9)

C1	O1	Cu1	119.37(7)	C6	C7	C2	119.49(10)
O1	C1	C2	115.81(9)	C5	C6	C7	120.57(11)
O2	C1	O1	125.47(10)	C4	C3	C2	120.62(11)
O2	C1	C2	118.68(10)	C3	C4	C5	120.18(11)
O4	C8	C7	118.88(9)	C4	C5	C6	119.73(11)

[1]+X,3/2-Y,1/2+Z; [2]3/2-X,3/2-Y,1/2-Z; [3]3/2-X,+Y,1-Z

Table S6. Hydrogen Atom Coordinates (Å×10^4) and Isotropic Displacement Parameters (Å^2×10^3) for **III**.

**Atom**	***x***	***y***	***z***	**U(eq)**
H1SA	1349	6765	836	37
H1SB	3146	6676	1312	37
H6	6903	5336	882	21
H3	5340	5436	4444	20
H4	5710	4467	3629	25
H5	6469	4414	1842	25
H1A	600(20)	6866(9)	4079(16)	25
H1B	160(30)	6818(8)	2775(17)	25
H1C	1760(30)	6808(9)	3491(16)	25
H1D	1070(30)	7346(9)	3360(20)	25

Table S7. Vibrational Spectra of Alkaline *o*-Phthalatodicuprates and Their Assignment

**I**		**II**		**III**		Assignment
Raman	IR	Raman	IR	Raman	IR	
	3552 m		3561 s			
		3532 w	3542 m		3538 s	
3518 w	3514 s					ν (H_2O)
3463 w	3465 s					
	3435 sh				3423 m	

		3403 w	3400 s			
		3334 w	3273 w	3259 w		
	3236 w	3198 w				
		3150 w		3190 w	3207 s	
			3105 w		3136 w	
3079 w						
3070 s		3075 s		3071 s		2
	3065 w	3063 m	3061 w	3059 s	3059 s	20
			3047 w	3054 w		ν (NH_4^+)
3033 w	3039 w	3033 w		3035 w		
	2990 w	3002 w	3012 w	3002 w		7b
			2994 w		2992 w	
	2961 w		2960 w			
					2865 m	
	1996 w					
	1965 w		1956 w			
	1934 w		1923 w			
	1860 w		1853 w			
			1819 w			δ (H_2O)
	1707 br			1726 w		
1653 w	1660 w	1679 w				
	1640 s		1629 m		1630 w	ν $(C=O)_{ip}$
	1615 s		1613 s			
1609 m		1609 w		1610 w	1610 m	ν $(C=O)_{oop}$
1592 s	1593 s	1599 s	1595 s	1594 w	1594 sh	
1584 s				1582 w	1588 s	8
1570 m	1573 s	1577 w	1573 s		1577 sh	

1561m				1565	1568 s	
		1538 w	1550		1549 sh	
	1490 w	1499 m	1493 m		1493 m	
1492 m	1487 m			1486 m	1486 m	19b
				1472 w	1475 w	
1444 w	1445 w	1452 w	1449 w	1449 w		
		1434 m	1426 s		1423 s	
1409 s	1403 m			1402 s		19a, δ (NH_4^+)
	1376 m	1378 w		1379 w	1376 s	
1368 m	1365 m	1369 m	1368 s	1363 w		
		1353 w	1355 s			
1298 w	1298 w	1298 w	1294 w	1300 w	1295 w	14
					1275 w	
1265 w	1262 w	1270 sh	1258 w	1252 w	1259 w	ν (C-O)
			1208 vw	1206 w		3
		1172 s	1170 w	1175 m		9a
1165 s	1161 w	1166 w			1168 w	
1153 w		1155 w	1152 w	1154 s	1158 m	
1148 w			1146 sh		1148 m	7a
1143 w	1144 m					
1085 w	1086 s	1085 w	1088 w	1087 w	1088 m	18a
					1077 w	
		1043 s				18b
1038 s	1035 w	1039 w	1036 W	1036 s	1036 w	
999w	1000 w					5
967 w	970 w	980 w				17b
			947		948	

896 w	890					γ (OH)
862 w	864	869 m	866 s		866 s	ρ (H_2O)
		854 w			852 sh	
836 vs	836	837 s	835 s	836 vs	835 s	
828 w	823 w					
		812 m	813 w	808 sh	811 m	1
798 w	799 W	798 m	797 w		798 m	
		788 w				
767 w	770 s					
		752 w	753 m		754 s	11
723 w	718 s	723 w	721 m		723 s	
711 w						
698 w	704 s	699 w	700 m	695 w	699 s	4
	680 - ?					
661 m	660 m	661 m	661 m		658 s	δ (CO_2)
	653 w	651 sh		653 m		
600 m	604 w			602 m		
		592 m	586 w			ω (H_2O)
	575 s	572 w		578 w	585 w	
562 w		558 w				9b
		552 w				
	539 w					
			519 w		513 br	
	505 m					ω (H_2O)
	488 w					
462 w	459 m	456 w	461 w	471 w	464 m	
			450 m		449 m	

435 w				438 s	437 w	
	426 w			431 w		16a
		420 w			412 w	
398 m	404 w	401 w	401 w	401 w	401 w	
354 w	356 m	366 m	361 m	374 w	369 w	6a
	337 w	340 w	341 m		342 w	16b
	317 w		319 w		318 w	ν_a (Cu-O)
305 w		307 m		306 w		ν_s (Cu-O)
	295 m		291 m	289 w		6b
276 w	277 sh	271 m	272 m	271 m	268 w	τ (CO)
269 w	265 m	260 w	258 sh	260 sh	259 w	
234 s				234 m	237 w	ρ (CO_2)
218 w		219 sh			223 w	17b
		202 s	206 w		202 w	
					198 w	ρ (CX)
	194 w				191 w	
179 m	177 w		177 w	176 w	180 w	17a
	165 w		169 w		170 w	
157 m	155 w		151 w		156 w	10b
	148 w					
141 w			141 w		143 w	15
					131 w	
118 w	115 w		118 w			τ (CX)
	91 w		99 w			

Notations: ν – stretching, δ – bending, ρ – rocking, ω – wagging, τ – twisting, γ – out-of-plane vibration; intensities: vs – very strong, s – strong, m – medium, w – weak, sh – shoulder, br – broad. Wilson's notation was used for ring vibrations.

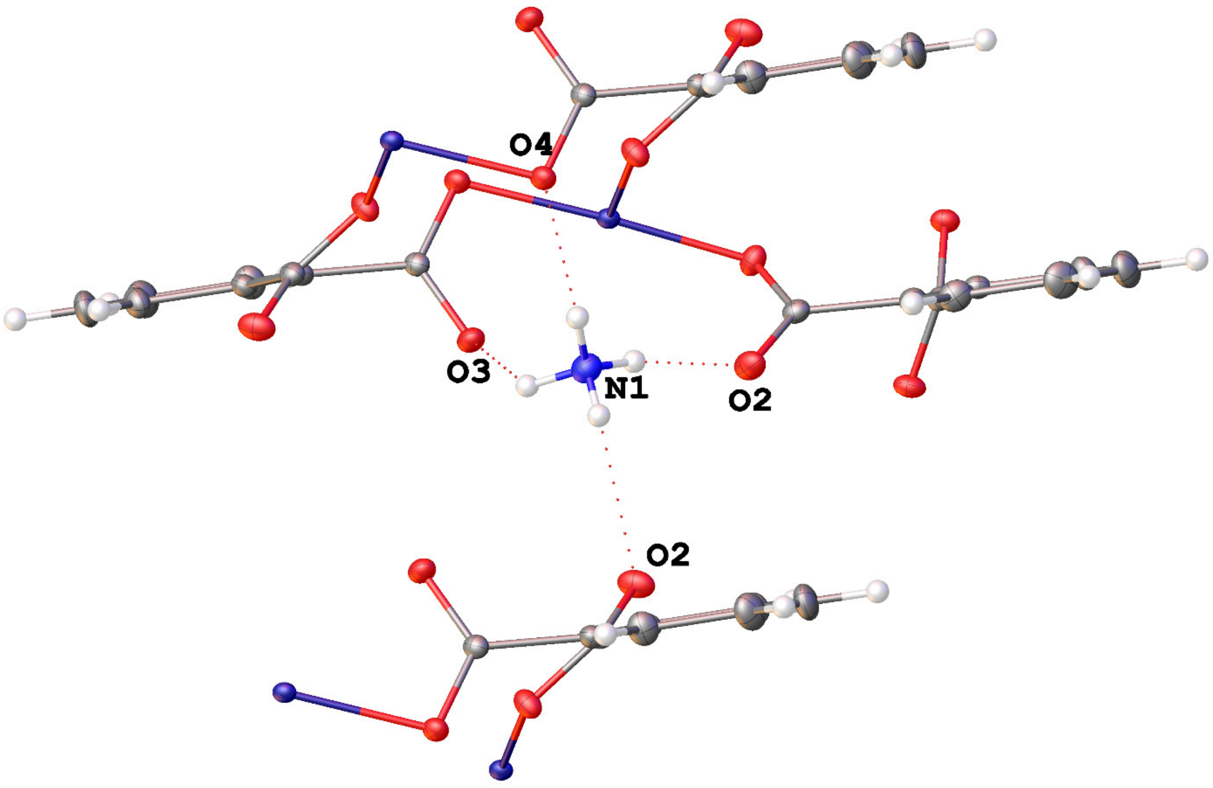

Figure S1. Ammonium ion interaction with phthalate ions in **III**.

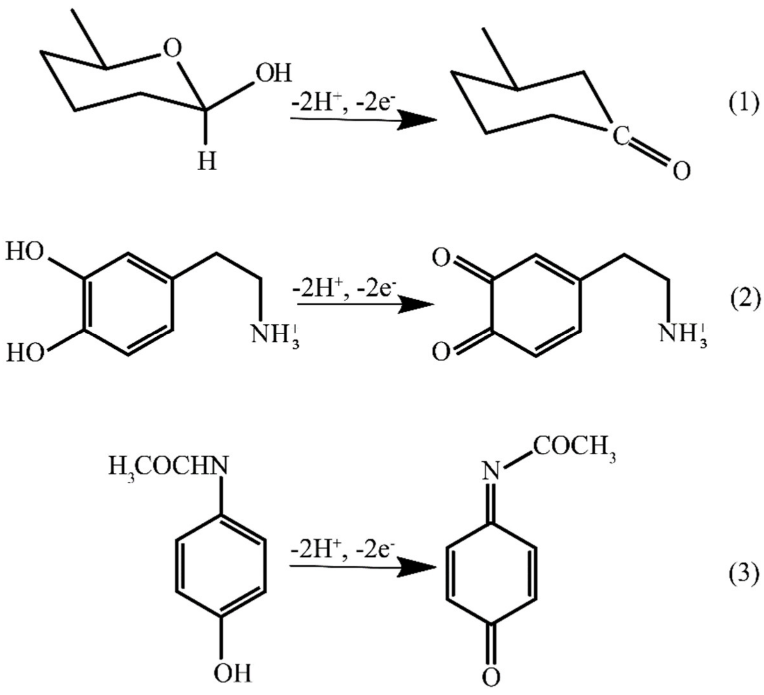

Figure S2. Chemical transformations of (1) Gl, (2) DA, and (3) AP during the electrooxidation
